# Roles of Surgery in the Treatment of Patients With High-Risk Neuroblastoma in the Children Oncology Group Study: A Systematic Review and Meta-Analysis

**DOI:** 10.3389/fped.2021.706800

**Published:** 2021-10-13

**Authors:** Yingyi Qi, Jianghua Zhan

**Affiliations:** ^1^Graduate College, Tianjin Medical University, Tianjin, China; ^2^Department of General Surgery, Tianjin Children's Hospital, Tianjin, China

**Keywords:** high-risk neuroblastoma, resection, induction chemotherapy, meta-analysis, surgery

## Abstract

**Purpose:** Neuroblastoma is the most common extracranial solid tumor in children, and most patients are at high risk when they are initially diagnosed. The roles of surgery and induction chemotherapy in patients with high-risk neuroblastoma have been a subject of much controversy and debate. The objective of the current study was to assess the roles of surgery in high-risk neuroblastoma.

**Method:** The review protocol was prospectively registered (PROSPEROID: CRD42021253961). The PubMed, Embase, Cochrane, and CNKI databases were searched from inception to January 2020 with no restrictions on language or publication date. Clinical studies comparing the outcomes of different surgical ranges for the treatment of high-risk neuroblastoma were analyzed. The Mantel–Haenszel method and a random effects model was utilized to calculate the hazard ratio (95% CI).

**Results:** Fourteen studies that assessed 1,915 subjects met the full inclusion criteria. Compared with the gross tumor resection (GTR) group, complete tumor resection (CTR) did not significantly improve the 5-year EFS [*p* = 1.0; HR = 0.95 (95% CI, 0.87–1.05); *I*^2^ = 0%], and the 5-year OS [*p* = 0.76; HR = 1.08 (95% CI, 0.80–1.46); *I*^2^ = 0%] of patients. GTR or CTR resection had significantly better 5-year OS [p = 0.45; HR = 0.56 (95% CI, 0.43–0.72); *I*^2^ = 0%] and 5-year EFS [*p* = 0.15; HR = 0.80 (95% CI, 0.71–0.90); *I*^2^ = 31%] than subtotal tumor resection (STR) or biopsy only; however, both CTR or GTR showed a trend for more intra and post-operative complications compared with the STR or biopsy only [*p* = 0.37; OR = 1.54 (95% CI, 1.08–2.20); *I*^2^ = 0%]. The EFS of the patients who underwent GTR or CTR at the time of diagnosis and after induction chemotherapy were similar [*p* = 0.24; HR = 1.53 (95% CI, 0.84–2.77); *I*^2^ = 29%].

**Conclusion:** For patients with high-risk neuroblastoma, complete tumor resection and gross tumor resection of the primary tumor were related to improved survival, with very limited effects on reducing intraoperative and postoperative complications. It is necessary to design strong chemotherapy regimens to improve the survival rate of advanced patients.

**Systematic Review Registration:** https://www.crd.york.ac.uk/PROSPERO/, PROSPEROID [CRD42021253961].

## Introduction

Neuroblastoma originating from the adrenal medulla or sympathetic ganglion is the most common extracranial solid tumor in children. It is most common in the abdomen (75%), followed by the mediastinum (20%) and the neck (5%) (1). The Children Oncology Group (COG) classified neuroblastoma patients as low-risk, medium-risk, or critical based on age at diagnosis, biological characteristics of the tumor including MYCN status and genomic segmental aberrations, International Neuroblastoma Pathology Classification (INPC), tumor DNA index, and tumor stage as defined by the International Neuroblastoma Staging System (INSS) (2). Notably, however, groups from different regions of the world do not use a consistent approach to classify patient risk. Investigators of major national and international cooperative groups from North America (COG), Europe (SIOPEN-R-NET), Germany (GPOH), and Japan (JANB/JINCS) developed the International Neuroblastoma Risk Group (INRG) classification and staging systems (INRGSS) using data from over 8,000 neuroblastoma patients internationally. The INRG classification system assigns neuroblastoma patients to 1 of 16 pretreatment risk groups based on INRGSS, age, histologic category, grade of tumor differentiation, MYCN amplification, and 11q aberration. Children with high-risk neuroblastoma account for approximately half of all patients diagnosed with neuroblastoma. Combined studies indicate that long-term survival rates of children with high-risk neuroblastoma are currently ~40–50% (3). Current therapy for high-risk neuroblastoma (HRNB) consists of combinations of intensive multi-agent induction chemotherapy, surgery, radiation, myeloablative consolidation therapy with stem cell rescue and transplantation, 13-cis retinoic acid, and immunotherapy (4). The prognosis of children in the high-risk group is poor, and the long-term disease-free survival rate is <50%. There is an urgent need to improve the prognosis of neuroblastoma, especially in refractory or high-risk patients, and increase the tumor resection rate and reduce the risks associated with surgery (5).

Because of the small number of published studies and their heterogeneity, many systematic reviews have reported inconsistent results. The current investigation was an updated meta-analysis on the survival rate and complications associated with surgery for neuroblastoma. More specifically, a systematic review and meta-analysis was performed to identify the effects of complete tumor resection (CTR), gross tumor resection (GTR), subtotal tumor resection (STR), and biopsy only (BX) on overall survival (OS), event-free survival (EFS), and complications. Whether induction chemotherapy has any positive effects on survival rates was also investigated.

## Materials and Methods

### Search Strategy

The review protocol was prospectively registered (PROSPEROID: CRD42021253961). A systematic review was performed in accordance with the Preferred Reporting Items for Systematic Reviews and Meta-Analyses (PRISMA) guideline (6). The search was conducted in the major electronic databases of PubMed, Embase, and CNKI, we had no restrictions on the language of the article.

Taking PubMed as the example, the keyword of the search was “(((surgery[Title/Abstract]) OR (resection[Title/Abstract])) AND (neuroblastoma[Title/Abstract]))NOT(olfactory)”; “Neuroblastoma/surgery”[Mesh]; (neuroblastoma[Title]) AND (surgical[Title]), (surgery[Title/Abstract]) AND ((induction chemotherapy) OR (neoadjuvant chemotherapy)).

When several studies reported findings for the same patients, the most recent or most complete study was chosen.

### Inclusion Criteria

Studies were included on the basis of the following criteria: (1) patients who were diagnosed as high risk of NB according to Children's Oncology Group (COG) risk stratification; (2) the patients underwent surgery, or only underwent pathological puncture, or received induction chemotherapy before the operation; (3) the scope of the operation was determined; (4) the follow-up period of the patient was at least 5 years, and the OS or EFS of the patient was involved; and (5) there is a record of the number of intraoperative or postoperative complications.

**Figure 1:**
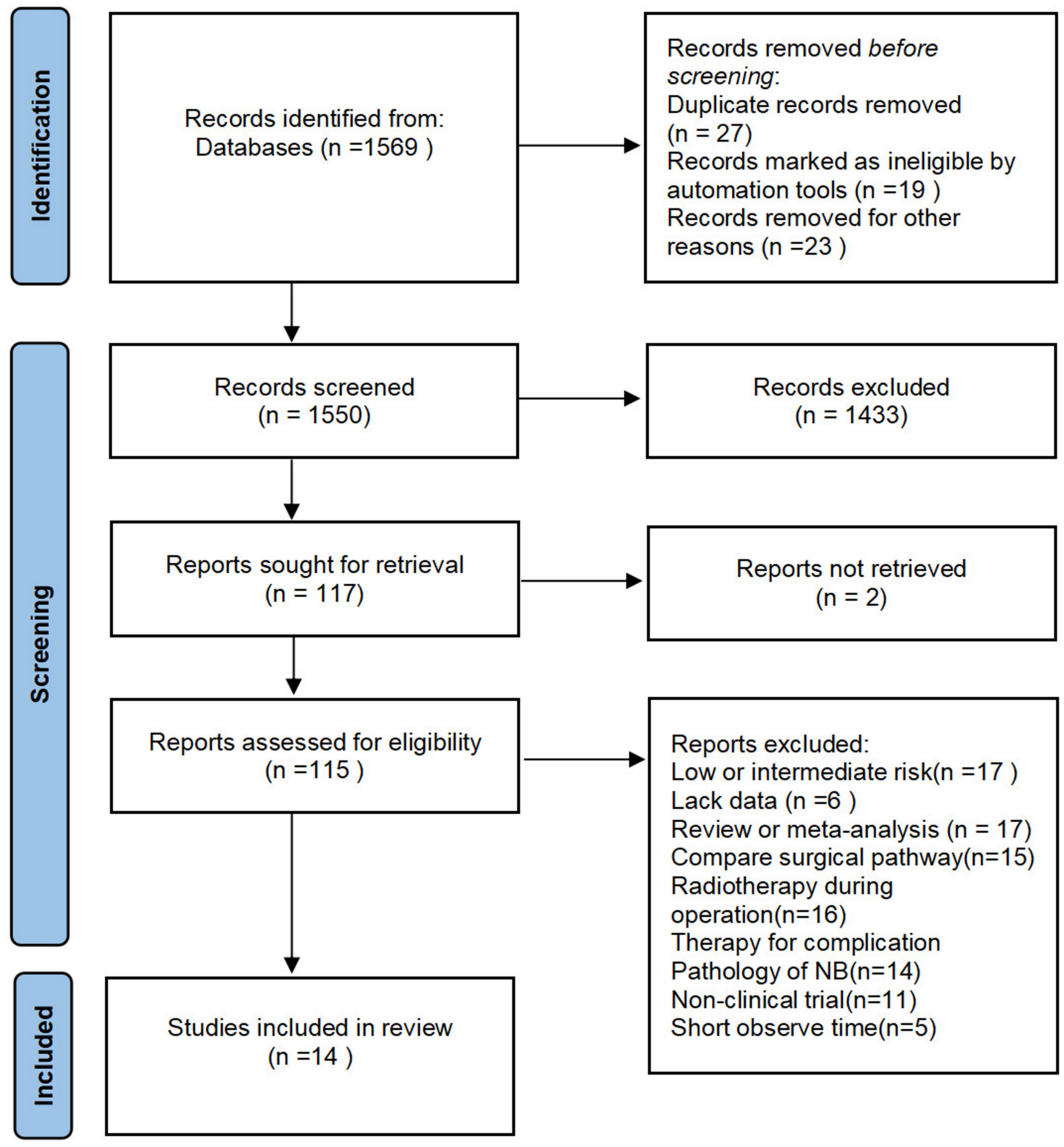
Flow diagram.

We excluded some articles in which the follow-up time was not enough and the survival rate was calculated by combining patients with GTR and patients with STR or biopsy only. The study would be excluded if the percentage of the tumor removal was not clearly stated in the article. A few of the studies, including patients with low or mediate risk neuroblastoma, also were excluded (Figure 1).

### Definitions

For the meta-analysis, we collected those who carried out CTR (complete tumor resection), GTR (gross tumor resection), STR (subtotal tumor resection), or BX (biopsy only), and those who accepted the induction chemotherapy before the operation. “CTR” represents macroscopic total removal of all visible tumor and nearby abnormal lymph nodes, “GTR” represents resection of tumor leaving a minimal macroscopic residue or removal of more than 90 or 95% of the visible tumor, “STR” represents removal of more than 50% but <90 or 95% of the visible tumor. “induction chemotherapy” means that systemic chemotherapy prior to local treatment, such as surgery or radiotherapy, is intended to shrink the mass and kill invisible metastatic cells early enough to facilitate subsequent surgery, radiotherapy, and so on (7).

### Data Extraction

We reviewed all titles and abstracts to determine eligibility and retrieve articles. The following information was extracted according to a fixed protocol: study design, geographical location, stage, sample size, group number, number of complications (Table 1). The long-term survival rate was defined by the 5-year OS and EFS.

**Table 1:** Characteristics of included studies.

**References**	**Country**	**Stage**	**Sample size**	**Group number**		**No. of complication**
Vollemer et al. (8)	Germany	IV^a^:40	40	50–90%/BX^b^:11 100/>90%:29		50–90%/BX:2 100/>90%:15
Adkins et al. (9)	USA	II:1 III:72 IV:466	539	Initial surgery 100%:120; >95%:60 <95%:51;BX:213	Best surgery 100%:210>95%: 115 <95%:74;BX:69	Best surgery 100%:60>95%:44 <95%:27
Englum et al. (10)	USA	NA^c^	87	100%:33;>90%: 23 <90%:21;BX:7		
von Allmen (24)	USA	NA	220	100%/>90%:154 <90%/BX:66		100%/>90%:37 <90%/BX:13
			84	100%/>90%:62 <90%/BX:22		
Li et al. (11)	China	NA	96	100%:39;>90%:23 <90%:10; BX:24		
Castel et al. (12)	Spain	IV:98	98	Initial surgery 100%:4; >90%:1 <90%:1;BX:74	Best surgery 100%:39;>90%:21 <90%:11;BX:5	
Mcgregor et al. (13)	USA	NA	124	>95%:7 <95%:3;BX:114	>95%:83 <95%:5; <50%:9	
Simon et al. (14)	Germany	IV:278	278	Initial surgery 100%:17;>90%:2 <90%:12; BX:246	Best surgery 100%:152;>90%:68 <90%:17;BX:37	
De Ioris et al. (15)	Italy	NA	58	>95%/100%:45 <95%/BX:13		
Yeung et al. (16)	China	IV:34	34	100%:24;>95%:6 <95%:4		
Koh et al. (17)	China	IV:19	19	100%:9;>95%: <95%:5		
von Allmen et al. (18)	USA	NA	76	100%:48;>90%:1250–90%:10;BX:6		
Salim et al. (19)	UK	III:13 IV:56	63	>95%:21; <95%:19 BX:23		
Tsuchida et al. (20)	Japan	IV:102	102	100%/ <100%:75 BX:10		

a *Staging on the basis of International Neuroblastoma Staging System (INSS) criteria*.

b *The percentage represents the degree of tumor resection*.

*BX, biopsy only*.

*NA, not available*.

### Validity Assessment

The quality of included studies was accessed independently by using the Newcastle–Ottawa Quality Assessment Scale. The scale was comprised of three factors: patient selection, comparability of the study groups, and assessment of outcome. A score of 1 was awarded for each item if the standard was completely met, a score of 0.5 was awarded if the standard was partially met, and a score of 0 was awarded if it was not met or if it was unclear whether it was met. The total score for each study was then calculated, a score of >6 indicated a high-quality study, a score of ≥3 and ≤ 6 indicated a median-quality study, while a score of ≥0 and ≤ 2 indicated a low-quality study (21).

### Statistical Analysis

Hazard ratios (HRs) with 95% CIs were calculated according to calculate lnHR and its variance by survival curves. Odds ratios (ORs) with 95% confidence intervals (CIs) were calculated based on the reported numbers of patients and events. The significance of the pooled OR/HR was evaluated by a *Z*-test, and a *p*-value of <0.05 was considered significant. Statistical heterogeneity among studies was evaluated by *I*^2^ and Q statistics. *I*^2^ values of <50% correspond to low levels of heterogeneity sensitivity and subgroup analyses were used to explore potential causes of heterogeneity (22). A *p*-value of <0.05 was considered significant for heterogeneity. Publication bias was assessed with funnel plots (23).

## Results

Fourteen studies that assessed 1,915 subjects were included in the meta-analysis. The sample size ranged from 40 to 539 issues (Table 1). All of the studies were published in or after 1992. Their validity scores are shown in Table 2. Seven articles are of high quality, nine articles are of medium quality, and low-quality articles were not included in this meta-analysis. von Allmen et al. (24) analyzed two groups of patients, one of these groups were determined by local surgeons' assessment, the other was determined by imaging central review. Adkins et al. (9), Castel et al. (12), and Simon et al. (14) separately recorded the survival rate of patients with the initial operation at diagnosis and delayed operation after induction chemotherapy.

**Table 2:** The score of included studies.

**References**	**Selection**	**Comparability**	**Outcome**	**Total**
Vollemer (8)	2.5	0	2.5	5
Adkins et al. (9)	3	1	2.5	6.5
Englum et al. (10)	3	1	2.5	6.5
von Allmen (24)	3	1	2.5	6.5
Li et al. (11)	3	1	2	6
Castel et al. (12)	2.5	0	2.5	5
Mcgregor et al. (13)	3	1	2.5	6.5
Simon et al. (14)	3	1	2.5	6.5
De Ioris et al. (15)	3	1	2.5	6.5
Yeung et al. (16)	3	1	2	6
Koh et al. (17)	2.5	1	2.5	6
von Allmen et al. (18)	3	1	2	6
Tsuchida et al. (20)	2.5	1	2	5.5
Salim et al. (19)	2.5	1	2.5	6

### Meta-Analysis Findings

**Figure 2:**
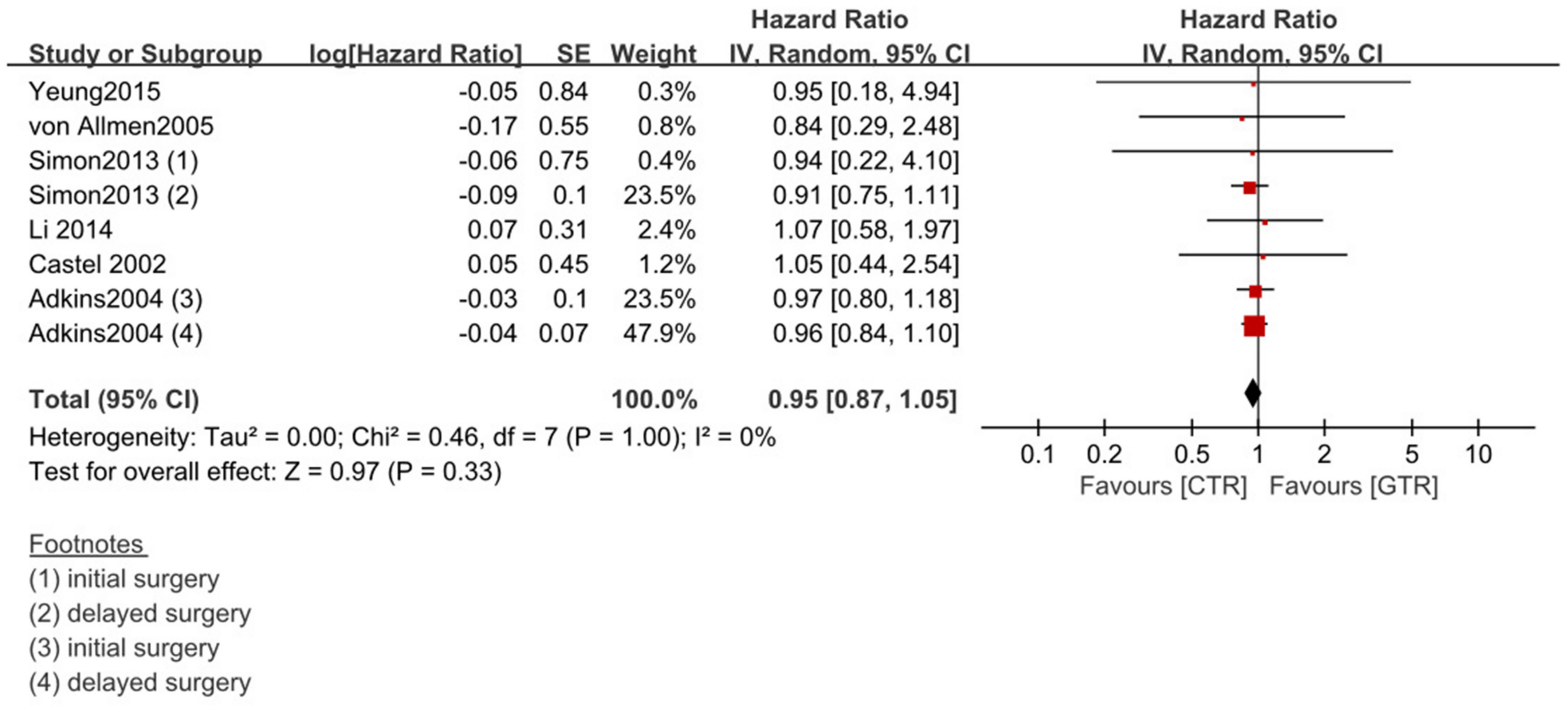
Effect of complete tumor resection (CTR) vs. gross tumor resection (GTR) on 5-year event-free survival (EFS) in high-risk neuroblastoma patients. Weights are from Mantel–Haenszel random effects analysis. Hazard ratios are shown with 95% confidence intervals.

**Figure 3:**
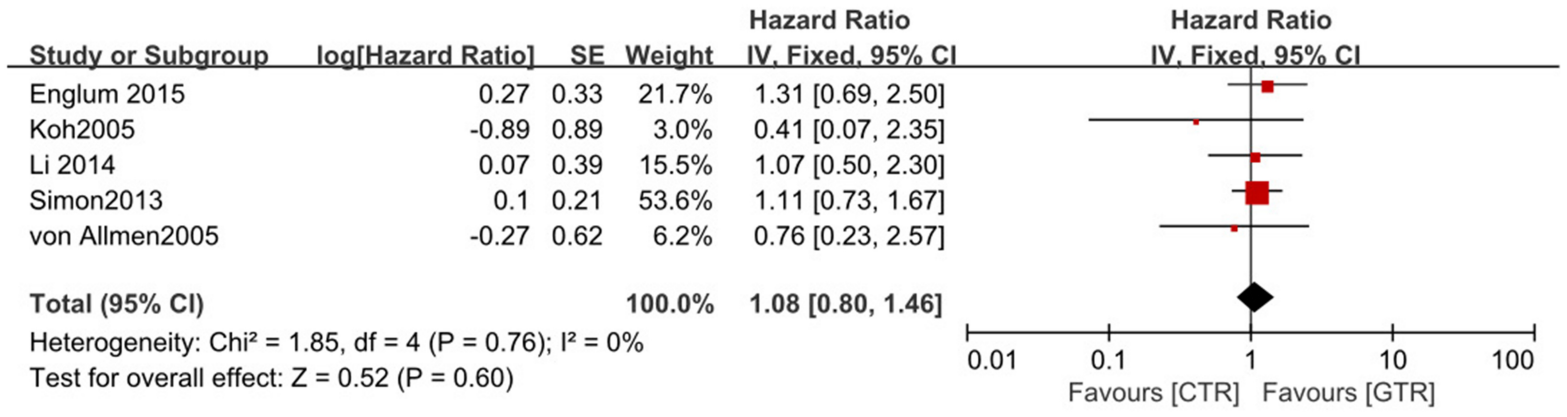
Effect of CTR vs. GTR on 5-year overall survival (OS) in high-risk neuroblastoma patients. Weights are from Mantel–Haenszel random effects analysis. Hazard ratios are shown with 95% confidence intervals.

**Figure 4:**
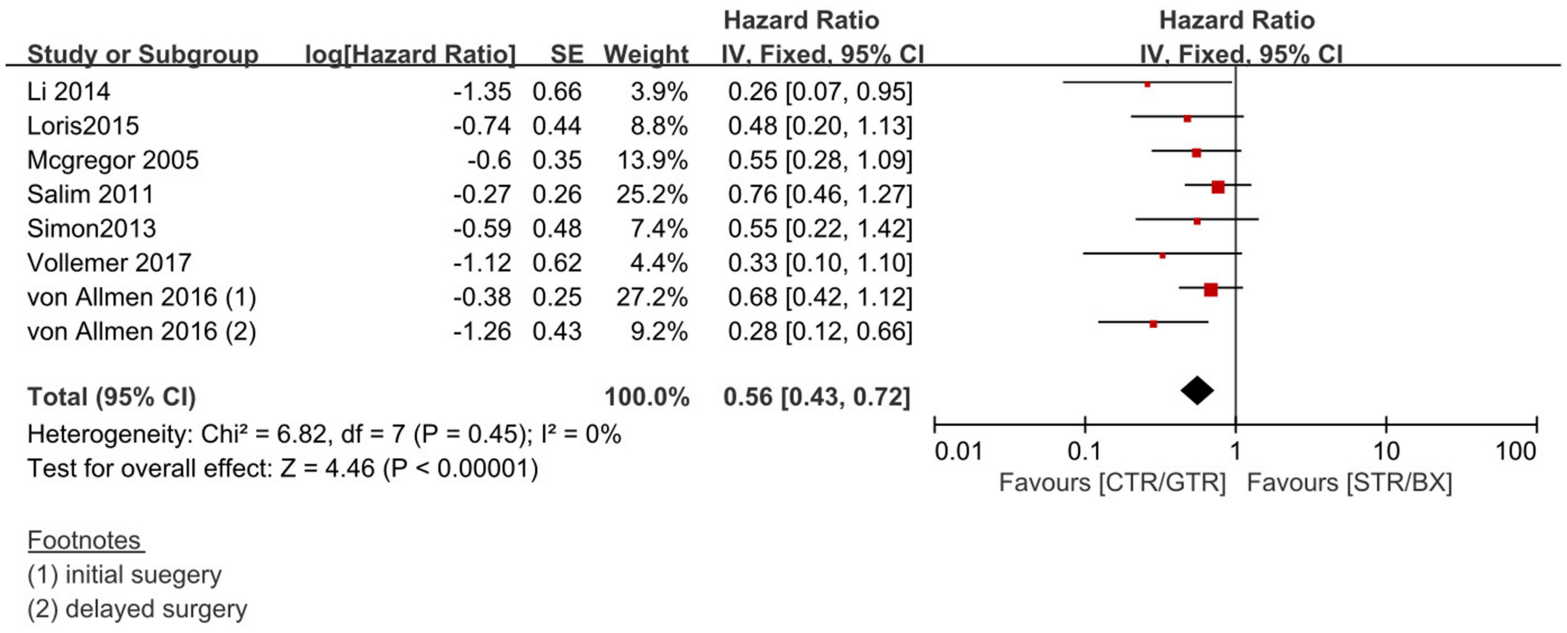
Effect of CTR/GTR vs. subtotal tumor resection (STR)/biopsy only (BX) on 5-year OS in high-risk neuroblastoma patients. Weights are from Mantel–Haenszel random effects analysis. Hazard ratios are shown with 95% confidence interval.

**Figure 5:**
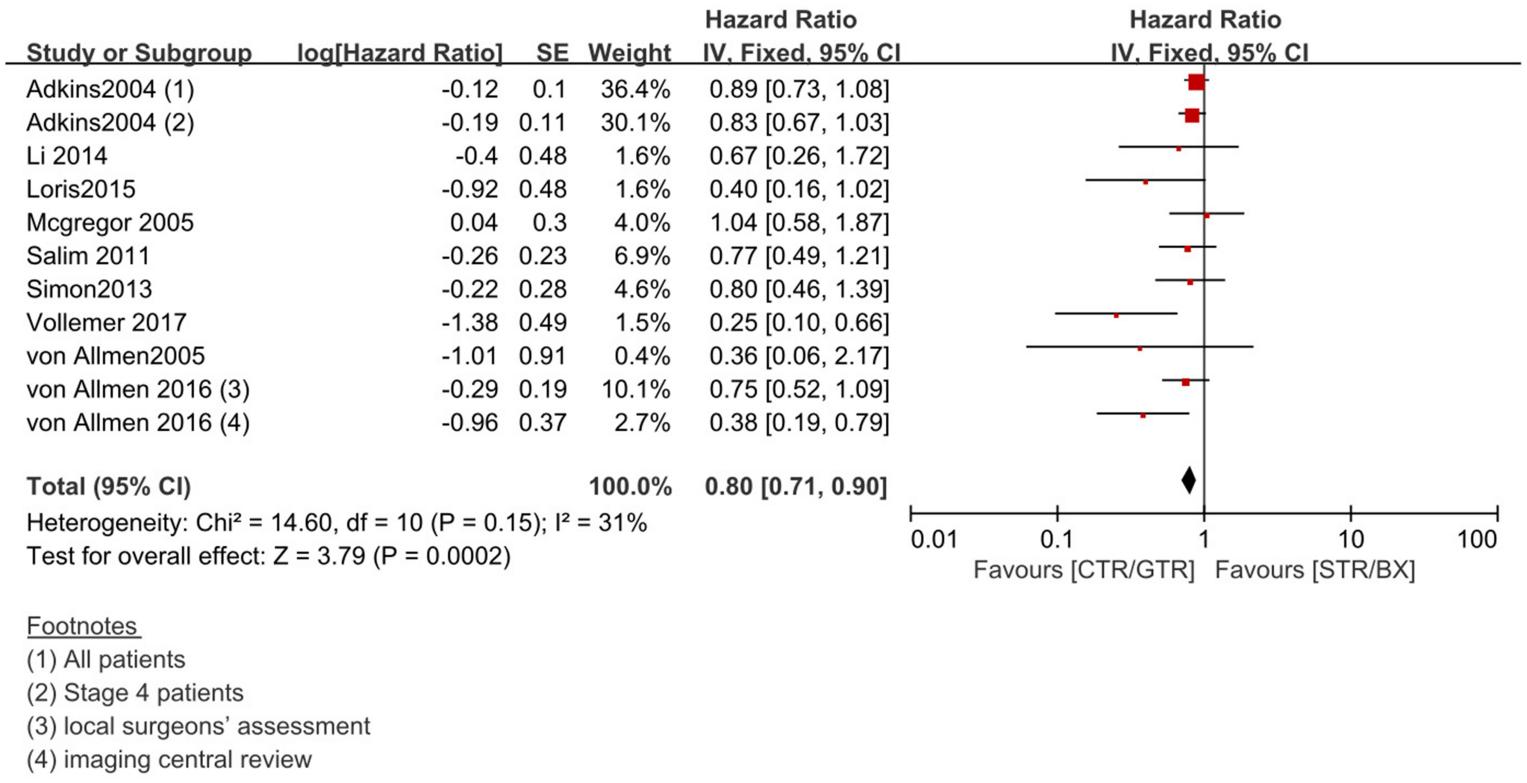
Effect of CTR/GTR vs. GTR/BX on 5-year EFS in high-risk neuroblastoma patients. Weights are from Mantel–Haenszel random effects analysis. Hazard ratios are shown with 95% confidence intervals.

**Figure 6:**
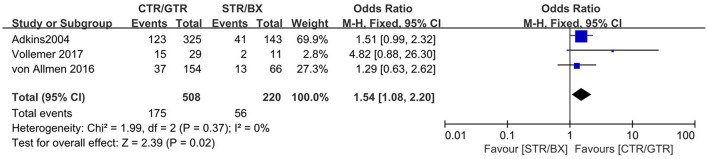
Complications of CTR/GTR vs. STR/BX during or after surgery in high-risk neuroblastoma patients. Weights are from Mantel–Haenszel random effects analysis. Odds ratios are shown with 95% confidence intervals.

Compared with the gross tumor resection (GTR) group, complete tumor resection (CTR) did not significantly improve the 5-year EFS [*p* = 1.0; HR = 0.95 (95% CI, 0.87–1.05); *I*^2^ = 0%] (Figure 2) and 5-year OS [*p* = 0.76; HR = 1.08 (95% CI, 0.80–1.46); *I*^2^ = 0%] of the patients (Figure 3). GTR or CTR resection had significantly better 5-year OS [*p* = 0.45; HR = 0.56 (95% CI, 0.43–0.72); *I*^2^ = 0%] (Figure 4) and 5-year EFS [*p* = 0.15; HR = 0.80 (95% CI, 0.71–0.90); *I*^2^ = 31%] (Figure 5) than subtotal tumor resection (STR) or biopsy only; however, both CTR or GTR showed a trend for more intra and post-operative complications compared with the STR or biopsy only [*p* = 0.37; OR = 1.54 (95% CI, 1.08–2.20); *I*^2^ = 0%](Figure 6). The EFS of the patients who underwent GTR or CTR at the time of diagnosis and after induction chemotherapy were similar [*p* = 0.24; HR = 1.53 (95% CI, 0.84–2.77); *I*^2^ = 29%] (Supplementary Figure 1).

### Subgroup Analysis

We repeated the meta-analyses on the basis of year (>2010 or <2010) and quality (high or moderate; Table 3), and the result is consistent with that of the primary meta-analysis.

**Table 3:** Subgroup analysis assessing hazard ratio (HR).

**Subgroups**		**EFS:CTR vs. GTR**	**OS:CTR vs. >GTR**	**EFS:CTR/GTR vs. STR/BX**	**OS:CTR/GTR vs. STR/BX**
Years	>2010	HR = 0.93 [0.77, 1.12] I^2^ =0% *P* = 0.97	HR = 1.15 [0.83, 1.57] *I*^2^ = 0% *P* = 0.89	HR = 0.65 [0.52, 0.81] *I*^2^ = 29% *P* = 0.2	HR = 0.56 [0.43, 0.74] *I*^2^ = 12 *P* = 0.34
	<2010	HR = 0.96 [0.86,1.08] *I*^2^ = 0% *P* = 0.99	HR = 0.62 [0.23,1.69] *I*^2^ = 0% *P* = 0.57	HR = 0.86 [0.75,0.99] *I*^2^ = 0% *P* = 0.68	Only one study
Quality	High	HR = 0.95 [0.86, 1.05] *I*^2^ = 0% *P* = 0.97	HR = 1.6 [0.82, 1.64] *I*^2^ = 0% *P* = 0.66	HR = 0.82 [0.72, 0.93] *I^2^* = 25% *P* = 0.24	HR = 0.54 [0.72, 0.93] *I*^2^ = 25% *P* = 0.24
	Moderate	HR = 1.02 [0.66, 1.58] *I*^2^ = 0% *P* = 0.98	HR = 0.88 [0.48, 1.61] *I*^2^ = 0% *P* = 0.59	HR = 0.62 [0.43, 0.90] *I^2^* = 36% *P* = 0.2	HR = 0.60 [0.39, 0.94] *I*^2^ = 42% *P* = 0.18

### Publication Bias

In the funnel diagram of the GTR or CTR vs. STR or biospy only (Supplementary Figure 2) group and CTR vs. GTR group (Supplementary Figure 3), grouping of studies at the apex of the plot suggested that larger studies with higher patient numbers are more likely to have been included. The lack of studies gathered at the base of the plot suggests a paucity of publications of smaller sample size.

## Discussion

Neuroblastoma is the most common extracranial solid tumor in children, and most patients are at high risk when they are initially diagnosed. The roles of surgery and induction chemotherapy in patients with high-risk neuroblastoma have been a subject of much controversy and debate. The objective of the current study was to assess the roles of surgery in high-risk neuroblastoma.

### Effects Between Gross Tumor Resection and Complete Tumor Resection on Event-Free Survival and Overall Survival

A systematic review by Zwaveling et al. (25) investigated the current status of surgical treatment of neuroblastoma. Of the 20 studies included in their analysis, only 4 explicitly compared survival in patients who underwent CTR with survival in patients who underwent GTR. In 2 of these studies, CTR yielded more favorable results than GTR, whereas in the other 2 there were no significant differences in survival. The authors concluded that a true comparison of the effects of surgery on survival based on previous studies was severely hampered. In the current meta-analysis, seven studies compared survival rates in GTR and CTR groups. Li et al. (11) and Simon et al. (14) reported that survival was similar in the CTR and GTR groups. Although, Adkins et al. (9), Castel et al. (12), Yeung et al. (16), Koh et al. (17), and von Allmen et al. (24) did not perform statistical comparisons between CTR and GTR groups, they all followed up the two groups of patients postoperatively and plotted survival curves. Compared with GTR, in addition to an increased extent of resection, lymph node dissection around the primary site or even primary organ resection achieved the purpose of complete resection in some groups. In the current meta-analysis CTR had little effect on the survival rate of neuroblastoma patients compared with GTR. Therefore, it is not necessary to pursue complete resection with lymph node dissection or removal of the primary organ.

### Effects Between Gross Tumor Resection/Complete Tumor Resection and Subtotal Tumor Resectioon/Biopsy Only on Event-Free Survival and Overall Survival

Nine studies in the current meta-analysis compared OS and EFS in patients treated between CTR/GTR and STR/biopsy only, but the results were not consistent. Vollemer et al. (8) reported that children who underwent GTR or CTR have significantly better OS and EFS than children who underwent partial resection. Englum et al. (10) and Li et al. (11) reported clear trends toward improved OS associated with CTR. von Allmen et al. (24) reported that >90% resection was associated with better EFS than <90% resection. McGregor et al. (13), De Loris et al. (15), von Allmen et al. (18), and Salim et al. (19) reported that the extent of best operation had no significant effect on EFS or OS. The reason for the difference in these four studies may be that the MYCN gene—which promotes tumor cell proliferation and inhibits apoptosis and differentiation—is evidently closely related to neuroblastoma occurrence and development, possibly limiting conclusions pertaining to these outcomes. Last, the location of the primary tumor, the level of experience of the surgeon, and the treatment compliance of the patient after the operation affects recovery. Although the above-described studies did not support CTR or GTR, the results of the current meta-analysis were mainly positive.

### Intraoperative and Postoperative Complications

In the present analysis, five reports described intraoperative and postoperative complications. Unexpectedly, Vollemer et al. (8), Adkins et al. (9), and Salim et al. (19) reported that complications were unrelated to the extent of resection. In the current meta-analysis, CTR and GTR were associated with increased complications, possibly because in many situations complete resection may have been abandoned after one or more complications occurred during the operation or after the initial operation.

### Induction Chemotherapy

Induction chemotherapy is now thought to make surgery easier. The most commonly used induction chemotherapeutic regimen (developed at the Memorial Sloan-Kettering Cancer Center) includes dose-intensive cycles of cisplatin and etoposide alternating with vincristine, doxorubicin, and cyclophosphamide. COG investigators added topotecan to this induction regimen on the basis of data indicating anti-neuroblastoma activity in cases of relapse. European protocols have utilized OPEC/COJEC regimens, which include vincristine, cisplatin, etoposide, and cyclophosphamide in OPEC, with additional carboplatin for COJEC (26). In the current meta-analysis, three studies mentioned the prognosis of induction chemotherapy. McGregor et al. (13) reported that patients who underwent GTR or CTR at the time of diagnosis had higher predicted 5-year survival than patients who had GTR or CTR after induction chemotherapy. Survival rates were also compared in 17 patients with primary surgical resection and 75 patients with delayed surgical resection by Tsuchida et al. (20), but there was no statistically significant difference in survival rate between these two groups. Adkins et al. (9) and Simon et al. (14) reported the survival rates of patients who underwent complete resection before and after chemotherapy, but they did not conduct statistical analysis. In the current analysis there was no significant difference in EFS between patients who underwent initial surgery and those in whom surgery was delayed. It is therefore necessary to design strong chemotherapy regimens to improve the survival rate of advanced patients.

### Association With Other Studies

Previous meta-analyses have drawn various conclusions depending on the types of control interventions used for comparison. Two of them are about the surgery method for NB. A systematic review by Yang et al. (27) published in 2018 included 18 studies. Although he also showed that the pooled effects of gross resection were significantly superior to other surgical options, the classification of the scope of operation was too general. In these included studies, the definition of gross tumor resection was different, and it had no effect on the postoperative and intraoperative complication rate. What is more, another study by Mullassery et al. (28) included 15 studies; the subjects of Mullassery are patients with stages III and IV of NB, and there are some patients with a low and moderate risk of NB, so the results deviated greatly. Even so, this study drew a conclusion that a clear survival benefit is shown for GTR or CTR over STR in stage 3 NBL only. Though some advantages can be demonstrated for GTR as defined by DFS in stage 4 NBL, GTR did not significantly improve OS in stage 4 disease. Considering the observed heterogeneity, this can be considered to be approximately beneficial to wider resection. Therefore, both articles have come to a similar conclusion with this meta-analysis, that is, removal of all tumors as far as possible can effectively improve the survival rate of patients. We also summarized the commonly accepted induction chemotherapy regimens to provide a more detailed reference for doctors to treat high-risk patients in the future.

### Limitations

The present meta-analysis had some limitations. A more precise analysis could have been conducted if individual patient data were available, enabling adjustment for age, sex, ethnicity, and geographical location. Different research institutions administer different chemotherapeutic drugs to patients; there is no unified standard for the evaluation of surgical tolerance, and biological heterogeneity affects clinical results. Immunotherapy and myeloablative therapy followed by autologous stem cell transplantation have yielded improved outcomes in collaborative trials, so larger and higher-quality trials are needed to confirm these conclusions.

## Summary

For patients with high-risk neuroblastoma, complete tumor resection and gross tumor resection of the primary tumor were related to improved survival, with very limited effects on intraoperative and postoperative complications. It is necessary to design strong chemotherapy regimens to improve the survival rate of advanced patients.

## Data Availability Statement

The original contributions presented in the study are included in the article/Supplementary Material, further inquiries can be directed to the corresponding author/s.

## Author Contributions

YQ contributed to the conception, design of the study, and drafting of the article. JZ contributed to revising the article critically for important intellectual content and contributed to the final approval of the version to be submitted. All authors contributed to the article and approved the submitted version.

## Funding

This study was funded by the Tianjin Health Bureau special grant (grant number: 14KG129) and the Tianjin Children's Hospital special grant (grant number: Y2020002).

## Conflict of Interest

The authors declare that the research was conducted in the absence of any commercial or financial relationships that could be construed as a potential conflict of interest.

## Publisher's Note

All claims expressed in this article are solely those of the authors and do not necessarily represent those of their affiliated organizations, or those of the publisher, the editors and the reviewers. Any product that may be evaluated in this article, or claim that may be made by its manufacturer, is not guaranteed or endorsed by the publisher.

## Supplementary Material

Supplementary Figure 1 EFS of the patients who underwent CTR or GTR tumor resection at the time of diagnosis and the patients who had CTR or GTR tumor resection after induced chemotherapy.

Supplementary Figure 2 Funnel plot for **(A)** 5-year OS in the comparison between CTR/GTR vs. STR/BX **(B)** 5-year EFS in the comparison between CTR/GTR vs. STR/BX tumor resection.

Supplementary Figure 3 Funnel plot for **(A)** 5-year OS in the comparison between CTR or GTR **(B)** 5-year EFS in the comparison between CTR or GTR.
