# Supplementary figures and images for: Roles of Surgery in the Treatment of Patients With High-Risk Neuroblastoma in the Children Oncology Group Study: A Systematic Review and Meta-Analysis
Source: Front Pediatr. 2021 Oct 13;9:706800. doi: 10.3389/fped.2021.706800 (PMC8548868; doi:10.3389/fped.2021.706800)

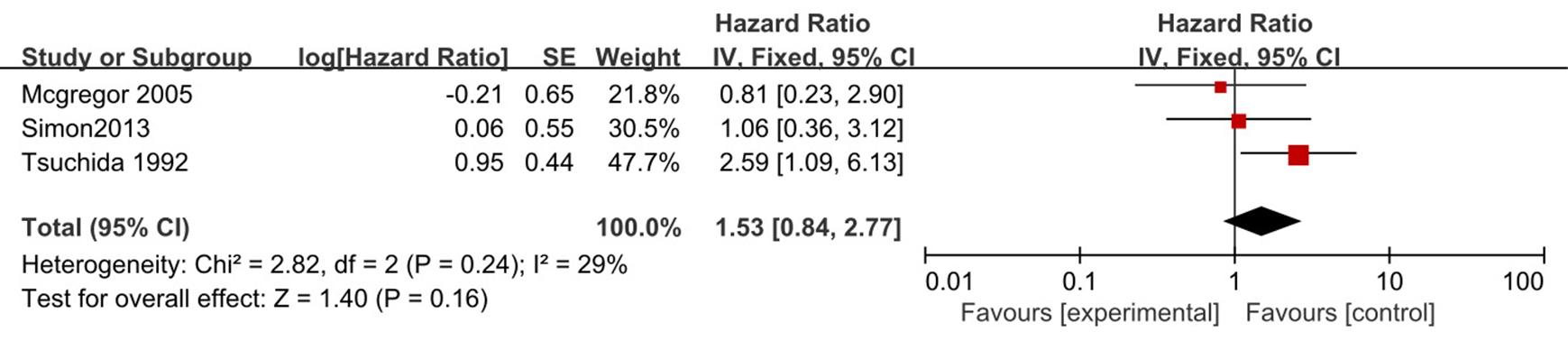

Supplement: Supplementary Figure 1 — EFS of the patients who underwent CTR or GTR tumor resection at the time of diagnosis and the patients who had CTR or GTR tumor resection after induced chemotherapy. [file Image_1.JPEG]

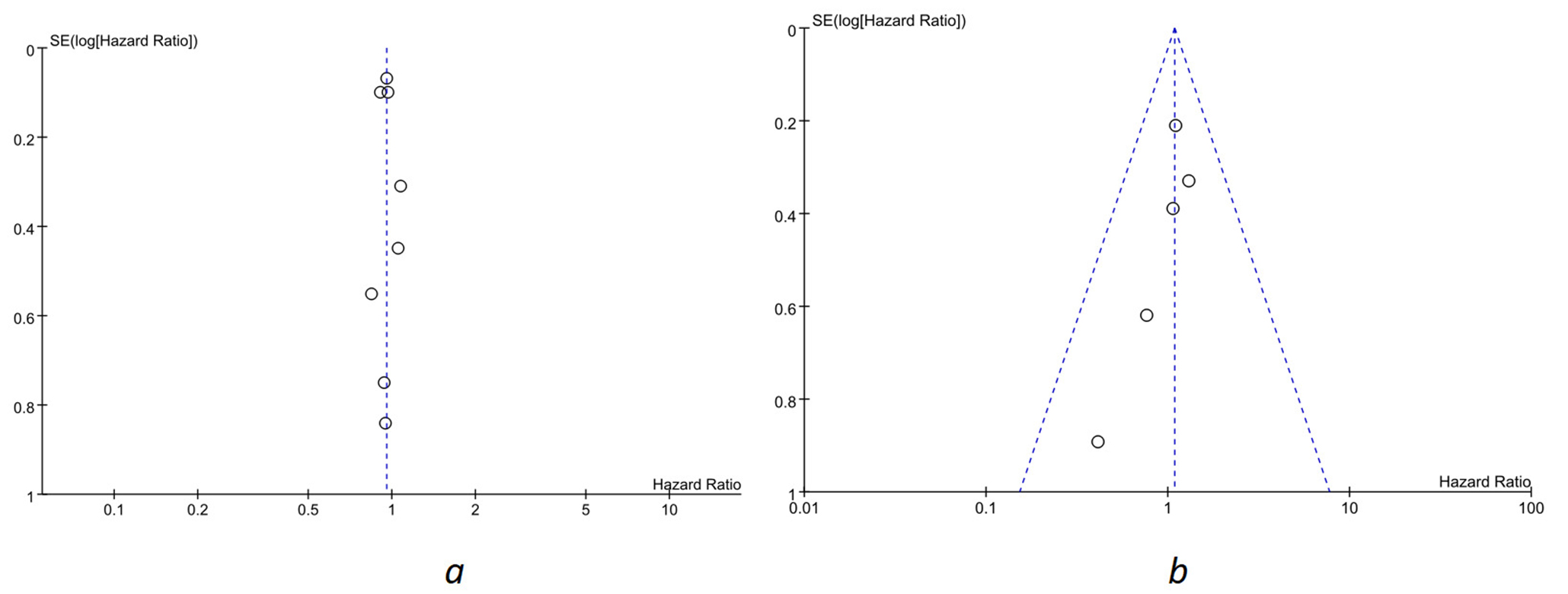

Supplement: Supplementary Figure 2 — Funnel plot for (A) 5-year OS in the comparison between CTR/GTR vs. STR/BX (B) 5-year EFS in the comparison between CTR/GTR vs. STR/BX tumor resection. [file Image_2.JPEG]

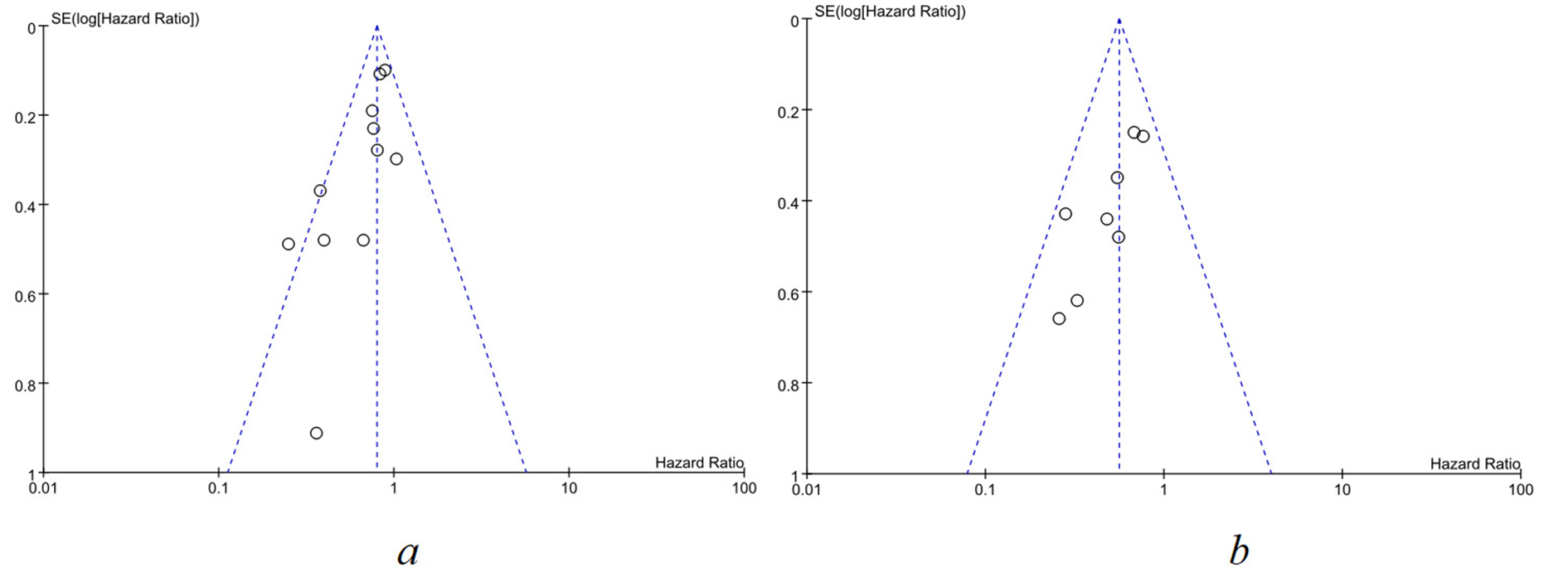

Supplement: Supplementary Figure 3 — Funnel plot for (A) 5-year OS in the comparison between CTR or GTR (B) 5-year EFS in the comparison between CTR or GTR. [file Image_3.JPEG]
